# Experimental Analysis of the Mechanical Response of Masonry Columns Partially Confined with PBO FRCM (Fabric Reinforced Cementitious Mortar) Composites

**DOI:** 10.3390/ma16134812

**Published:** 2023-07-04

**Authors:** Luciano Ombres, Francesco Campolongo, Marielda Guglielmi, Salvatore Verre

**Affiliations:** 1 Department of Civil Engineering, University of Calabria, Arcavacata di Rende, 87063 Cosenza, Italy; fracampo27@gmail.com (F.C.); marielda.guglielmi@unical.it (M.G.); 2 Faculty of Engineering, University E-Campus, via Isimbardi 10, 22060 Novedrate, Italy; salvatore.verre@uniecampus.it

**Keywords:** columns, composites, confinement, masonry, partial

## Abstract

An experimental investigation on partially PBO (short of Polyparaphenylenebenzobisthiazole) FRCM (Fiber Reinforced Cementitious Mortar) confined clay brick masonry columns has been conducted. Ten small-scale specimens measuring 445 mm high with a square cross-section of the 250 mm side have been tested under monotonic axial loading until collapse. Two columns were unconfined, while the remaining ones were confined with single-layer PBO FRCM jackets varying the geometric configuration along their height. The vertical spacing ratio *sf’*/*sf*, being *sf’* and *sf* the center-to-center and the net spacings between two consecutive jackets, respectively, was considered as the key parameter of the confinement configuration. The failure modes, stress–strain curves and peak axial stress and strain values are reported. The experimental results have been compared to the predictions of models found in the Italian guidelines CNR DT 215/2018 and the American ACI 549-R20 standards. The main aspects analyzed involved (i) the evaluation of the effectiveness of partial confinement on the mechanical response of columns, (ii) the definition of the mechanical and geometrical parameters that influence the structural response of partially confined columns, and (iii) the development of appropriate analytical models for the prediction of the resisting capacity of masonry columns partially confined with PBO FRCM.

## 1. Introduction

In the last few decades, composite systems consisting of fabric meshes embedded into inorganic matrixes are often used as a strengthening system for masonry structures. These composites, generally identified as Fabric Reinforced Cementitious Matrix (FRCM) or Textiles Reinforced Mortar (TRM), have good mechanical properties and exhibit excellent durability under different environments conditioning such as alkaline [1,2], hot water [3] and freeze/thaw [4]. They are easily applied, their workability is guaranteed over a wide temperature range, and they have excellent compatibility with the masonry substrate due to the inorganic nature of the matrices [5]. Generally, the matrices used are eco-friendly [6,7]. In FRCM/TRM composites, the main role of the fibers is to bear tensile stresses. On the other hand, the mortar has multiple responsibilities in keeping the fabric together, protecting it from the environment’s aggressive agents, administering the stress transfer from the substrate to the fabric and also providing rigidity to the composite system. Thus, the mortar properties have an influential role in determining the potential FRCM structural performance; consequently, in the case of poor-quality mortar, the effectiveness of the strengthening can be limited or even compromised. Among the various structural applications, the one still attracting the most interest from the scientific community is the confinement of masonry columns. Since the content of fibers used for the confining systems must conform to design requirements, full confinement is generally adopted. Indeed, masonry columns are usually fully wrapped with FRCM/TRM jackets along their whole height; an alternative confining solution is a discontinuous one, according to which columns can be wrapped with longitudinally spaced FRCM/TRM jackets. Discontinuous confinement provides an optimal design solution for masonry columns requiring a small strength increase. It is useful in historic buildings where less invasive intervention is required, and the breathability of masonry has to be guaranteed.

Several experimental and theoretical studies have been carried out in the recent past to analyze the performances of masonry columns confined with FRCM/TRM. Most of these studies relate to fully confined masonry columns. These include several previously reported studies on columns confined with different FRCM systems such as basalt–FRCM [8,9], glass–FRCM [10,11,12], carbon–FRCM [13,14,15], steel–FRCM [16,17,18], PBO–FRCM [19,20]. The influence of the number of confinement layers, the mortar grade, the confinement configuration (corner radius, overlapping, fiber orientation), and the load eccentricity has been investigated.

Basalt–FRCM confined masonry columns [8,9] were tested to evaluate the influence of the mechanical properties of the mortar. Ten half-scale FRCM-confined masonry columns were built using standard tuff bricks and lime mortar. The columns were reinforced with the same types of glass fiber fabric, varying the properties of the inorganic matrix, including different types of cement and pozzolanic additives. The properties of the inorganic mortar have a significant effect on the behavior of the FRCM confined masonry columns and should be carefully considered in the design of retrofitting strategies.

Thirty-four masonry columns confined with basalt textile reinforced concrete (BTRC) have been tested under concentric and eccentric compression loads varying the load eccentricity and the number of basalt textile layers [10]. It was observed an enhancement of the load-carrying capacity of confined columns and higher efficiency under eccentric loading.

Three different inorganic matrices have been used to confine poor-quality masonry columns tested under axial load [11]. It was evidenced as a proper grade of the matrix can significantly improve the structural performances of confined columns.

Uniaxial compression tests were performed on full-scale limestone masonry columns confined with glass and basalt FRCM systems [12]. Six strengthening configurations by combining external FRCM reinforcement with internal pultruded or steel bars have been adopted. It was found that (i) the columns exhibited similar behavior (glass and basalt grids had similar mechanical and geometrical properties), (ii) the internal reinforcement was active after the FRCM jacketing failed, (iii)the failure mode was similar in all columns and (iv) the mortar contributed to the effectiveness of the FRCM confinement.

Cross-section geometry (square and rectangular), textile type (carbon, basalt and glass–FRCM) and the amount of the jacket’s reinforcement have been varied in an experimental investigation on textile-reinforced mortar confined masonry columns [13]. It resulted that the increase of the cross-section aspect ratio reduced the effectiveness of the confinement, while an almost linear increase between the axial strength and the reinforcement ratio was observed for a given textile type. In addition, the coating of textiles led to superior performance amongst all textile types for all cross-section aspect ratios. The same parameters have been considered in an experimental investigation on short masonry columns confined with carbon textile-reinforced mortar [14]. It was evidenced that (i) the increase of the cross-section aspect ratio has a stronger influence on the deformability than in strength;(ii) for a cross-section ratio higher than 1.5, the increase of the number of layers does not improve further the strength and strain and (iii) in some cases premature failure due to debonding on the overlap occurred.

Clay brick masonry columns fully confined with Carbon–FRCM (C–FRCM) have been tested under both axial and eccentrical loads [15]. The strength of confined columns increases significantly (almost 80%) with respect to that for unconfined columns; the increase was more limited and strongly dependent on the confinement ratio in eccentrically loaded columns (20 and 43% for single layer and two-layers C–FRCM jackets, respectively). All tested columns exhibited a gradual and pseudo-ductile failure due to the rupture of the carbon textile.

Thirteen small-scale clay brick masonry columns confined using steel-FRCM, PBO FRCM and basalt–FRCM has been tested under axial loads [16]. The effect of confinement ratio (i.e., number of textile layers), load eccentricity, and position of overlap zones along the height of the columns were analyzed. It was determined that (i) all the used FRCM confining systems are effective in enhancing both the strength and ductility of masonry columns, (ii) the structural response of columns was different for each confinement system, and (iii) both the confinement ratio and the confinement configuration influence the effectiveness of the confinement.

SRG (steel-reinforced grout) jackets have been used to confine clay brick masonry columns tested under concentrical load. The varied parameters were the corner radius and the density of steel fibers [17]. It resulted in the axial capacity of the masonry columns increasing with both fiber density and corner radius.

A round-robin test to investigate the behavior of masonry columns confined with FRCM composites has been carried out [18]. Evaluation of the performance of different FRCM systems, including different types of fiber (glass and steel), types of masonry (tuff and clay brick masonry) and confining ratio (i.e., number of confining plies) to provide recommendations for the design of retrofitting strategies for masonry structures have been carried out. The strength and ductility gains were both evident and proportional to the number of plies, as well as the geometrical percentage of fiber. The efficiency of the confinement was more evident for a lower-strength reference column.

A wide number of experimental results on FRCM fully confined masonry columns have been assembled into a database [19]. Two strength models calibrated by applying best-fit techniques to the experimental results were proposed to estimate the compressive strength of masonry columns confined by FRCM composites. A critical review of experimental data on masonry columns confined with FRCMs allowed a new predictive model to be formulated [20]. The model was calibrated by taking into account that FRCMs could lead to the different behavior of the confined column depending on the type of fiber used.

The performance of masonry columns confined with composites under axial compression has been evaluated using three different confinement techniques, namely FRP (fiber-reinforced polymers), FRCM and steel grids or wire hooping [21]. Three sets of experimental databases have been developed and analyzed to appraise the performances of confined columns in relation to various parameters. It resulted that the confinement is more effective in low-strength masonry than in high-strength masonry. The study also showed that while the application of discontinuous FRP wrapping has been studied quite exhaustively, the application of discontinuous FRCM around masonry columns has not been well explored.

Studies and research on the structural performances of FRCM partially confined masonry columns are, in fact, very limited. At present, only results from clay brick masonry columns partially confined with FRCM jackets are available [22,23]. Half-scale masonry columns confined with PBO FRCM have been tested under axial compression [22]. The confinement was achieved by both fully and partially wrapping varying the number of plies (1, 2 and 3). The PBO FRCM confined columns have improved load-bearing capacity, ductility and energy dissipation. In addition, by increasing the number of FRCM layers and using full-wrapping of the columns, better confinement effectiveness can be achieved. A theoretical model based on existing proposals to simulate the behavior of FRCM-confined masonry columns under axial compression was developed. An experimental investigation of partially FRCM-confined masonry columns has recently been conducted [23]. The confining system (basalt–FRCM, SRG), the vertical spacing ratio (i.e., the ratio between the center to center and the net spacings between two adjacent FRCM strips) and the number of the confining ratio (i.e., 1, 2, and 3 confining layer) were varied parameters. Increasing the number of FRCM layers permits an increase in the peak load and deformation capacity of the columns. Moreover, for each vertical spacing ratio, the peak load decreases with the increase of the axial rigidity (i.e., with the increase of the fabric thickness) of the FRCM strips. Current design codes consider partial confinement of columns only when FRP composites [24] are used. In that case, the reduced effectiveness of the confinement is accounted for by means of a coefficient that takes into account the geometric configuration of the confinement. Conversely, the partial confinement of columns with FRCM composites is not considered [25].

In this context, an in-depth analysis of the mechanical behavior of masonry columns partially confined with FRCM becomes appropriate. Since this solution, as described above, is significant and particularly useful in many practical situations, it is essential to evaluate its effectiveness in terms of both strength and deformability. At the same time, it is essential to identify both geometric and mechanical parameters that influence the mechanical response of columns partially confined with FRCMs for the definition of appropriate predicting models. To this end, the results of an experimental investigation conducted using small-scale brick masonry columns partially confined with PBO FRCM are reported. Ten masonry columns 445 mm high and with a square cross-section 250 mm side were tested under axial load until failure. Two columns were unconfined (reference columns); the remaining height was confined with single-layer PBO FRCM jackets in varied geometric configurations. The vertical spacing ratio *sf’*/*sf*, being *sf’* and *sf,* the center-to-center net spacings between two consecutive jackets, respectively, was considered as the key parameter of the confinement configuration. Five different *sf* values were chosen, with a jacket’s width of 55 mm, resulting in five vertical spacing ratios: 0.0, 0.36, 0.51, 0.68 and 1 (*sf’*/*sf* = 1 and *sf’*/*sf* = 0 refer to unconfined and fully confined columns, respectively). The main aspects analyzed concern (i) the evaluation of the effectiveness of partial confinement on the mechanical response of columns, (ii) the definition of the mechanical and geometric parameters that influence the structural response of partially confined columns, and (iii) the development of appropriate analytical models for predicting the resisting capacity of masonry columns partially confined with PBO FRCM. Failure modes, stress–strain curves and peak axial stress values are reported. The experimental results have been compared to the predictions of models found in the Italian DT 215/2018 [25] and the American ACI 549.4R-20 [26] standards.

## 2. Materials and Method

### 2.1. Specimen Preparation

**Figure 1:**
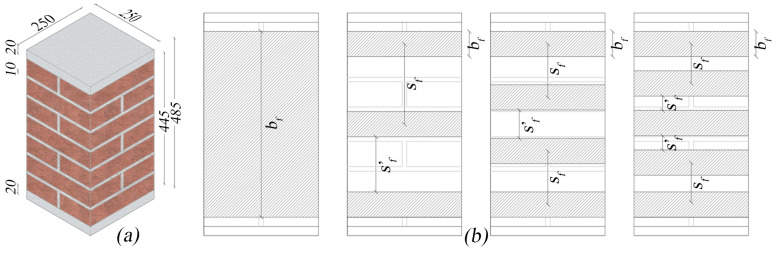
(**a**) Geometry of the specimens (dimensions are in mm) and (**b**) configuration of the confinement: from left to right ζ = 0 (fully wrapped), ζ = 0.68, ζ = 0.52, ζ = 0.36, respectively.

Tests on columns were conducted in two different series identified with the symbols (I) and (II). The considered masonry was clay brick with lime-based mortar made up. In order to impose the load on a uniform surface, capping was assessed by means of a stiff mortar layer at the top and bottom sides of the columns (20 mm-thick each). The installation of the FRCM jackets was executed after column wetting following a standard hand lay-up procedure [5]. Details of the geometrical configuration of the confined columns are illustrated in Figure 1. The main geometric parameter considered in the investigation is the vertical spacing ratio ζ = s_f_’/s_f_, whose values are given in Table 1. It should also be noted that in the confinement configurations considered in the investigation, the volume of fiber used varies with the vertical spacing ratio. If V_f0_ and V_f_ indicate the volume of fiber used in the fully confined and partially confined columns, respectively, the V_f_/V_f0_ ratio varies between 0.32 for ζ = 0.89 (CD1L-PBO 3 columns) and 0.60 for ζ = 0.37 (CD1L-PBO5 columns).

The designation *CX1L-PBOn (i)* is used to identify the specimens: *CX* indicates the type of confinement (*C* for continuous, *D* for discontinuous), 1L the single-layer reinforcement, PBO the type of fiber, n the number of stripes for each column and i = I or II the series of tests. The UCS label identifies the un-confined column. For all partially confined columns, the width of the PBO FRCM jackets was set equal to *bf* = 55 mm. As reported in Table 1, two specimens were tested for each confining configuration.

### 2.2. Binding Mortar and Clay Brick Units

Masonry columns have been built with commercial clay bricks of size 250 × 120 × 55 mm^3^. The average strength of clay bricks determined by standard compression tests on six bricks conducted in accordance with [27] was 28.30 MPa (CoV = 2%). All tests have been performed by a fluid dynamics machine with a load cell of 3000 kN. An electro-mechanics machine with a load cell of 100 kN has been used to perform tests in accordance with [28,29] on 40 × 40 × 160 mm^3^ prisms of the commercial patented mortars used to bind the clay units (Mortar 1) and to cap the columns (Mortar 2). Average compression (*f_c,mat_*) and flexural tensile (*f_b,mat_*) strength values and the coefficient of variation (CoV) values are reported in Table 2.

### 2.3. FRCM System

The PBO–FRCM adopted in this study, provided by [5], consists of a bidirectional unbalanced fabric mesh (a real weight of 70 g/m² and 18 g/m² in the longitudinal and transverse direction, respectively) embedded in a stabilized inorganic (cement-based) mortar. The spacing between PBO fiber yarns is 10 mm (equivalent thickness of 0.045 mm) and 20 mm (equivalent thickness of 0.012 mm) in the longitudinal and transversal directions, respectively. A ready-mix cement-based mortar was used (Mortar 3), whose mechanical properties are reported again in Table 2. The mechanical properties of dry textile were evaluated in accordance with [30] in terms of elastic modulus (*E_f_*), tensile strength (*f_fu_*) and ultimate strain (*ε_fu_*), and they were equal to 218 ± 12 GPa, 3409 ± 452 MPa and 1.56 ± 0.2%, respectively. Furthermore, a total of five direct tensile (DT) tests were carried out on PBO–FRCM coupons (50 × 6 × 500 mm^3^) to evaluate the mechanical response of the composite systems, according to Italian Guidelines [31]. The coupons were equipped with four PVC tabs at the ends having 50 × 2 × 100 mm^3^ dimensions in order to uniformly distribute the gripping force. All the specimens exhibited a three-phase tensile behavior, as expected when dealing with the gripping method.

In addition, the average values for the third phase in terms of tensile stress *σ_u_*, tensile strain and tensile modulus *E*_3_ are 1434.64 ± 254 MPa, 1.25 ± 0.35% and 114.72 ± 8 GPa, respectively. Thus, the efficiency factor assumed as the ratio between the tensile strength of the FRCM on the dry fabric was found to be equal to 42%.

### 2.4. Test Set-up

**Figure 2:**
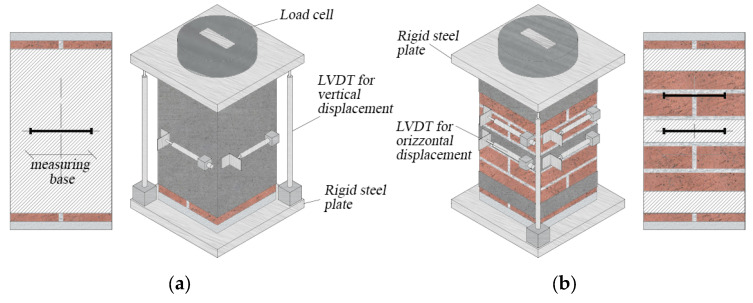
Scheme of the test set-up: continuous (**a**) and discontinuous (**b**) wrapping.

Figure 2 illustrates the test set-up of confined columns. All the tests were conducted in displacement control at a rate of 0.2 mm/min up to failure by a fluid dynamic machine with a load cell of 3000 kN. To uniformly apply the load, two 20 mm-thick steel plates have been positioned at the top and bottom of the columns (Figure 2). Two LVDTs (Linear Variable Displacement Transducers) have been located along the sides of two opposite corners to measure the vertical displacements.

Further LVDTs (with a 150 mm-gauge length) have been located at the mid-eight of the columns in the horizontal direction involving both the confined and unconfined areas (in case of discontinuous wrapping) in order to measure transversal displacements.

## 3. Results and Discussion

As shown in Table 1 in this analysis, only two columns were tested for each reinforcement configuration (i.e., for each vertical spacing ratio). It follows that the results obtained cannot be representative of the real behavior of masonry columns partially confined with FRCM; they provide useful indications for more extensive experimentation that should involve a larger number of samples for each type of masonry and reinforcement system (fiber type, matrix type, number of reinforcing fibers, etc.). To this end, a further experimental investigation on masonry columns partially confined with FRCM configurations designed in such a way as to keep the volume of reinforcing fiber constant for each of them has been planned by the authors. The tests are still in progress.

With the above in mind, the main results obtained in this analysis are described and discussed below in terms of failure modes, stress–strain curves, peak load, strains and ductility.

### 3.1. Failure Modes

**Figure 3:**
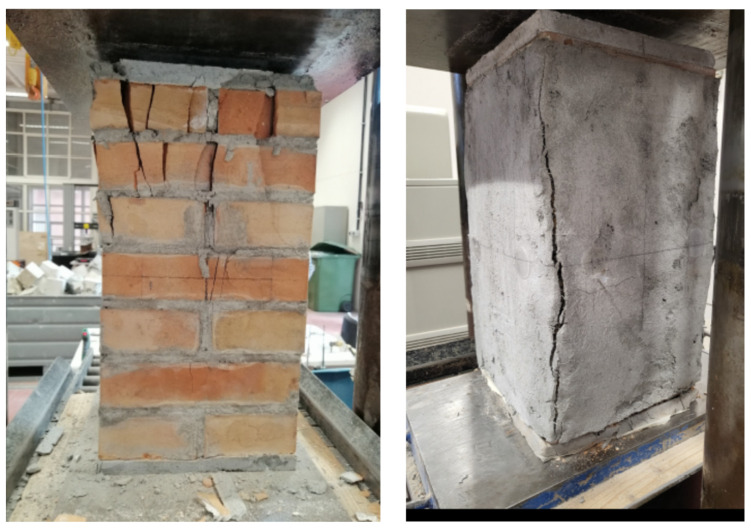
Unconfined and fully confined columns at failure.

The unconfined column failed when the crushing of the masonry occurred in one of the upper corners of the columns. A sub-vertical crack develops along the longitudinal direction (Figure 3), while numerous wide cracks form near the zone where the crushing occurred. At failure, the upper part of the corner was completely detached from the rest of the columns. Sporadic cases of material expulsion for the specimens UCS (II), which manifested a lower bearing capacity, have been observed.

**Figure 4:**
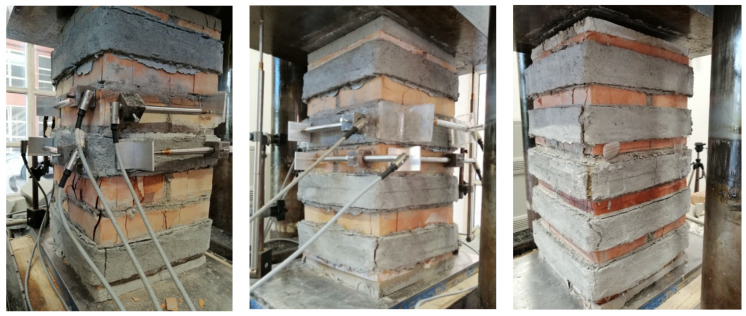
Partially confined columns at failure.

The collapse of the fully confined columns was dominated by the masonry crushing after the jacket detachment; a large vertical crack took place at the corner of the columns in correspondence with the overlap of the PBO jackets. The fabric remained un-cut, so it trended to slip within the matrix in agreement with the column shortening. The partially confined columns failed in a mode intermediate between that of the fully confined and unconfined columns. In fact, the FRCM-free regions were crushed (arching effect), while the confined ones induced the corner failure. Vertical cracks formed initially at the half height of the columns; then the crack pattern of the masonry substrate extended to the entire un-strengthened zone, while a series of vertical cracks began to appear on the composite strips, particularly in the overlap zone (Figure 4).

The FRCM strips exhibited multiple transversal cracks, with the corner cracks the most evident. Fabric sliding and detachment of jackets, probably caused by excessive stress concentration in the corners, are also observed in the post-peak behavior of the columns.

**Figure 5:**
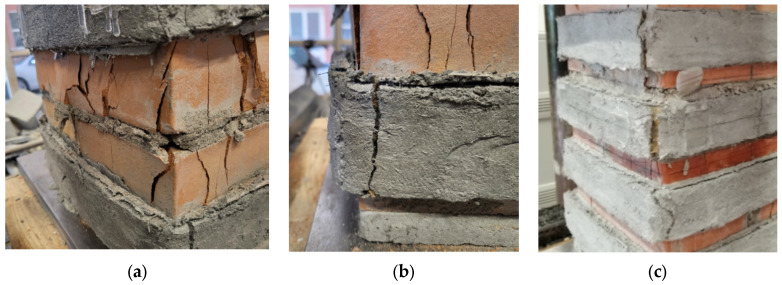
Details of the failures. (**a**) CD1L-PBO3 columns (**b**) CD1L-PBO5 columns (**c**) CD1L-PBO5 columns.

It was noticed that the higher the number of FRCM wraps (i.e., the lower the vertical spacing ratio), the more the failure was dominated by the corner effect. As illustrated in Figure 5a, in fact, for the highest values of the vertical spacing ratio (CD1L-PBO3 columns) at failure, widespread crumbling and the expulsion of brick chips in the unconfined zones were observed while in the confining jacket at the bottom of the column, a vertical cracks appeared in the proximity of the corner (Figure 5b). On the contrary, the failure of the CD1L-PBO5 columns, for which the lowest is the vertical spacing ratio, occurred for the detachment of the PBO FRCM jackets at the corners, while the masonry in the un-confined zones suffered limited damage (Figure 5c).

This different behavior is related to the occurrence of the arching effect in the unconfined zone of the columns. As well known, under high loads, the cracking pattern in the masonry extended with an increase in both the number and the width of cracks. An arch-like shape of vertical cracks formed, and the exterior part of the unconfined masonry was lost; the arching effect occurred in a form off parabola with an initial tangent slope of 45° in the area of unconfined masonry core of the confining jackets. However, the development of the arch needs an adequate distance between the transverse reinforcement, i.e., an adequate value of the vertical spacing ratio. Small values of the latter, in fact, limit the formation and the extensions of cracks in the unconfined masonry, avoiding the formation of arch-like vertical cracks.

This aspect needs deeper and more extensive investigations in order to evaluate the limit values of the vertical spacing ratio. For masonry columns confined with FRP, the Italian Guidelines provide the following limitation s’_f_ < d_min_/2 being d_min_ is the minimum value of the geometrical dimensions of the column’s cross-section. The limited available data for FRCM partially confined masonry columns does not allow us to formulate similar limitations.

**Figure 6:**
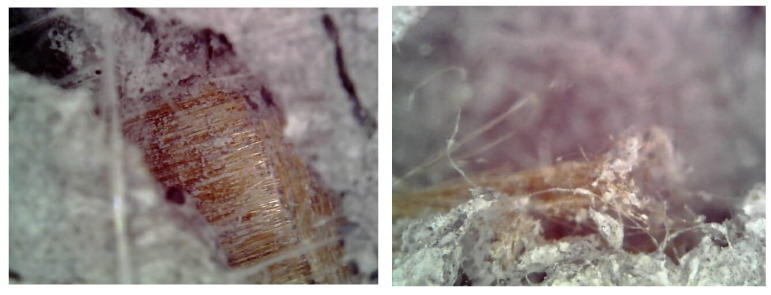
Details of failure at the microscopic level.

Moreover, microscopical images were taken within the mid-face and corner cracks, as reported in Figure 6, respectively. They revealed the trend of the PBO-yarn to slide with respect to the mortar matrix as visible by the un-damaged fibers having mortar dust on the exposed surface, while the rupture of the PBO-yarn stayed at the corner level.

### 3.2. Peak Load

Table 3 reports peak axial load values measured during tests for each specimen. Furthermore, the average value was computed for each series. Since two samples were tested per series, the average data was coupled with the scatter in percentage between the two recordings. In such a way, even if a set of minimum of three samples is needed to compute a statistically valid analysis, the eventuality of testing a further sample was taken under control by the %scatter being under a herein imposed fixed value equal to 10% (means negligible) [11].

The recorded values range between 1463.7 kN in the specimen fully wrapped and 737.16 kN in the un-confined column. As expected, the peak loads of partially confined columns are intermediate between the values obtained on fully wrapped columns and those obtained on un-confined columns.

The confinement ratio *ξ*, the ratio between the peak loads of confined columns and the average peak load recorded on unconfined columns, *P_UCS,avg_* = 765.45 kN, also reported in Table 3, is in the range of 1.49–1.91. It means that the partial confinement of the columns is effective for increasing the axial capacity of the masonry columns

**Figure 7:**
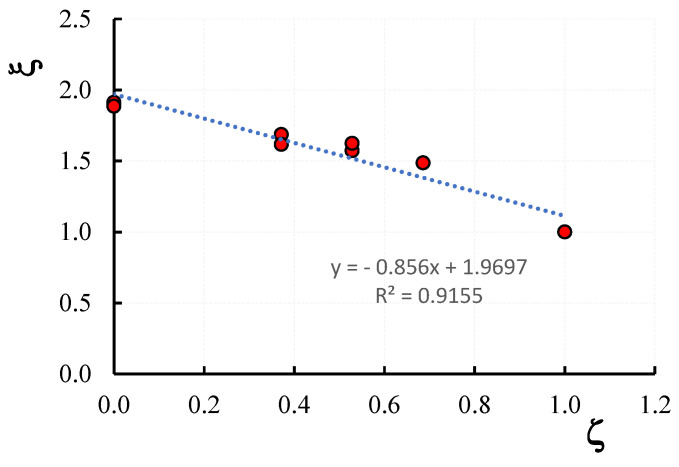
Confinement ratio, ζ, versus vertical spacing ratio ξ.

Figure 7 reports the variation of the confinement ratio ξ with the vertical spacing ratio ζ; it is evident that the ξ-ζ variation is well described by a linear relationship. As reported in Figure 7, the correlation coefficient of the trend line of the ξ-ζ law is indeed close to 1 (*R*^2^ = 0.9155). In other words, the more the strengthening scheme is distributed along the entire height of the columns, the higher the peak load obtained.

The analysis of the experimental results reported in Table 3 shows that for each couple of tested columns, the difference between the peak load values is lower than 10%; this result confirms that the peak load is not influenced by the cracking configuration, which, generally, can be different even for similar specimens.

An interesting result is that relating to the variation of the peak load with the volume of fiber reinforcement used in the confinement configuration. With respect to the values recorded for fully confined columns, the peak load values and the non-dimensional volumetric ratio V_f_/V_f0_ of partially confined masonry columns reduced an average of 22% and 68%, respectively, for the vertical spacing ratios of 0.89 (CD1L-PBO3 columns), 16% and 52%, respectively, for the vertical spacing ratio of 0.53 (CD1L-PBO4 columns), 13% and 13% respectively for the vertical spacing ratio of 0.37 (CD1L-PBO5 columns). Therefore, it seems possible to optimize the partial confinement configuration so as to significantly reduce the amount of reinforcing fiber with modest reductions in the resistant capacity. As such, in this analysis, the configuration corresponding to ζ = 0.53 yields a little different resistant capacity than that corresponding to fully wrapping with a 52% reduction in the amount of reinforcing fiber. However, further investigation is needed to confirm and generalize this outcome.

### 3.3. Stress-Strain Curves

**Figure 8:**
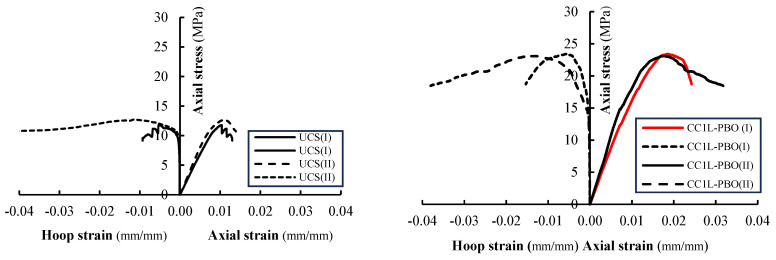
Axial stress vs. axial and hoop strain for unconfined and fully confined columns.

**Figure 9:**
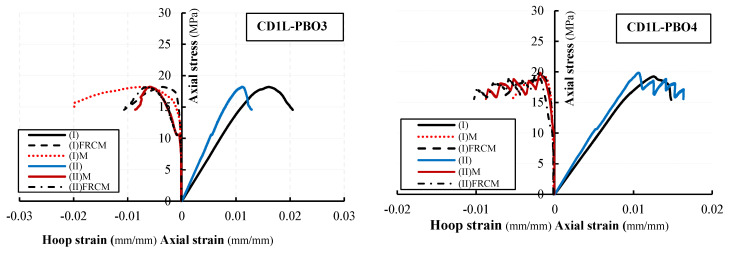

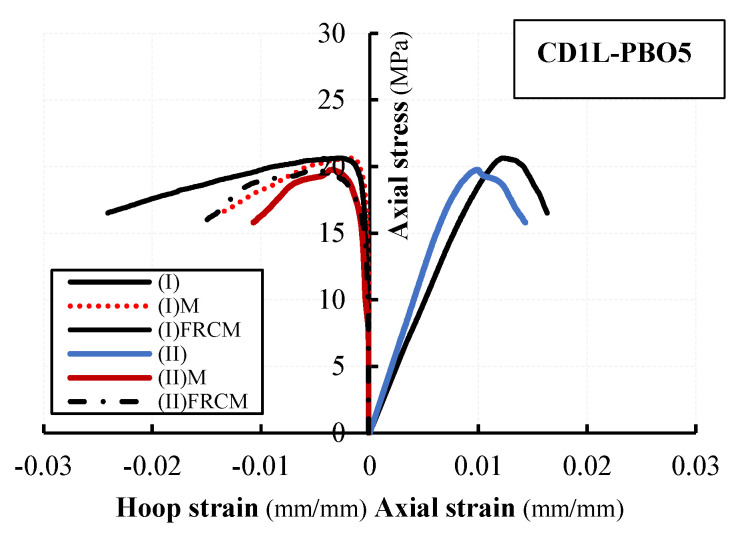
Axial stress vs. axial and hoop strain for partially confined columns.

The axial stress-axial strain and hoop strain curves evaluated from test results are illustrated in Figure 8 and Figure 9 for all tested columns. The axial stress values have been calculated as σ_v_ = *P*/*A* being *P* the applied load recorded by the load cell transducers and *A* the area of the column’s cross-section (250 × 250 mm^2^); axial strains were obtained by dividing the axial shortening (average of the vertical LVDTs readings) by the gauge length of the LVDTs. Hoop strain values have been determined by dividing the average of the measures obtained from the four horizontal LVDTs by the gauge length (150 mm).

Figure 8 and Figure 9 report the hoop strain values measured in the un-confined masonry (the curves are labeled with M) and those recorded in the confining jackets (the curves are labeled with FRCM). Figure 8 and Figure 9 evidence that the behavior of PBO FRCM—partially confined masonry columns can be described as a nearly linear branch until the peak stress is reached; a descending softening branch is, then, observed until failure occurs. Figure 8 and Figure 9 show that the values of hoop strains are very small in comparison with axial ones; they become significant only when confinement is activated.

**Figure 10:**
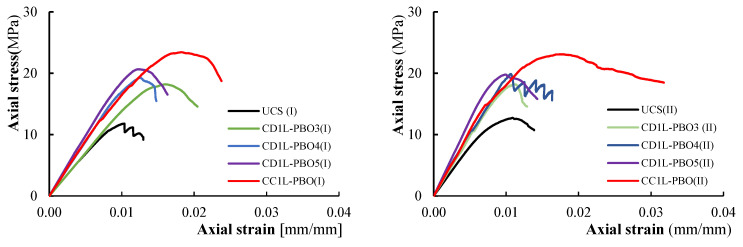
Axial stress-axial strain curves for tested columns.

The comparison of axial stress-axial strain curves varying the vertical spacing ratio is reported in Figure 10. For all tested confined columns, the initial linear behavior follows the behavior of the unconfined columns: as the axial stress approaches the masonry strength, the behavior of the confined columns becomes non-linear until the peak stress is reached. After the peak stress value, the behavior of confined, partially confined and unconfined columns changes significantly. Unconfined columns evidence a strong reduction of the stiffness and a fragile rupture (no softening branches are present in stress–strain diagrams). The behavior of the fully wrapped columns is described by a plateau in correspondence with the peak stress followed by an extended softening branch. For partially confined columns, a typical softening branch has been observed in the stress–strain diagrams. Different values of the ultimate axial strain have been measured in both fully confined and partially confined columns. The maximum recorded values of axial strain at peak stress and at the ultimate are reported in Table 3.

It was observed that strain values were different within similar specimens; in some cases, significant differences are found in the ultimate axial strain (24% for CD1L-PBO3 columns). This is an expected result; the strain values, in fact, are related to the axial stiffness of the specimens, which is reduced by the cracking pattern and can have significant variations even among similar specimens. The highest values of axial strains have been found in fully wrapped columns, while for partially confined columns, the measured strains are almost identical for the different reinforcement configurations (i.e., for the considered values of the vertical spacing ratio). This also means that for partially confined columns, the ultimate axial strains are not dependent on the volumetric ratio.

As shown in Figure 8 and Figure 9, the initial slope of the curves’ axial stress-axial strain for low values of the load is similar for all the tested columns (both unconfined and confined). This means that at this stage, the confinement is not active.

After the crack formation, the slope varies with the confinement configuration; this is due to the stiffness degradation caused by the cracking distribution. As previously described, after the attainment of the peak stress, a substantial change in the slope has been observed.

**Figure 11:**
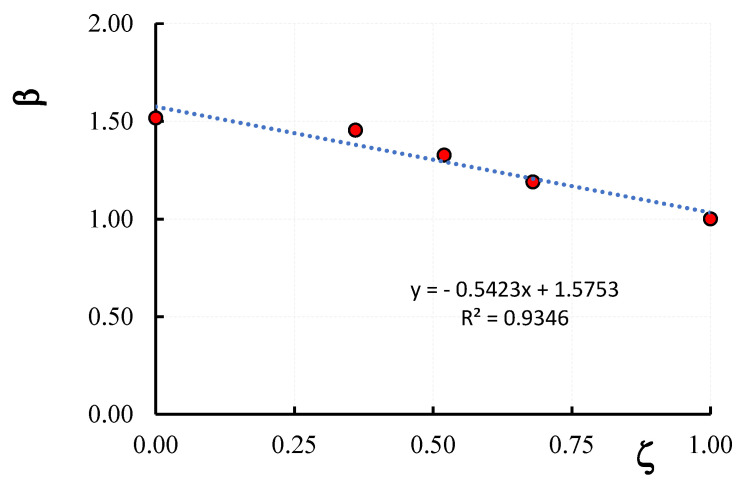
Variation of the elastic modulus of confined columns with the vertical spacing ratio.

The elastic modulus value in the tested columns, *E_mas_*, calculated by the slope of the axial stress-axial strain curves from 5 to 40% of the maximum axial stress, are reported in the last column of Table 3. The obtained results show that the elastic modulus value in the confined columns is higher than that in the unconfined ones. On average, the increase is 19%, 32.7%, 45.4% and 51.7% for ζ = 0.68, 0.56, 0.36 and 0, respectively. The variation between the ratio *β* = *E_confmas_*/*E_uncmas_* being *E_confmas_* and *E_uncmas,_* the elastic modulus of confined and unconfined masonry columns, respectively, and the vertical spacing ratio ζ, is illustrated in Figure 11. The equation of the *β-ζ* trend line is shown in the same figure; as can be noted, the *β-ζ* variation can be efficiently represented by a linear law (the correlation coefficient is *R*^2^ *=* 0.936).

On the other hand, with respect to the fully wrapped configuration, the elastic modulus (i.e., the stiffness) of the partially confined columns has been reduced. In detail, on average, reductions of 4.1%, 12.5% and 21.5% for ζ-values of 0.37, 0.53 and 0.89, respectively, were obtained. These reductions are related to the cracking patterns that occurred in confined columns; since cracking is more prevalent in the unconfined areas of the columns, the maximum stiffness reduction was achieved in columns where the unconfined areas are larger (CD1L-PBO3 columns). Relating the reduction in stiffness to the volume ratio, it was observed that even with large reductions in the amount of reinforcing fiber, the reduction in stiffness in the initial phase is small.

### 3.4. Ductility

**Figure 12:**
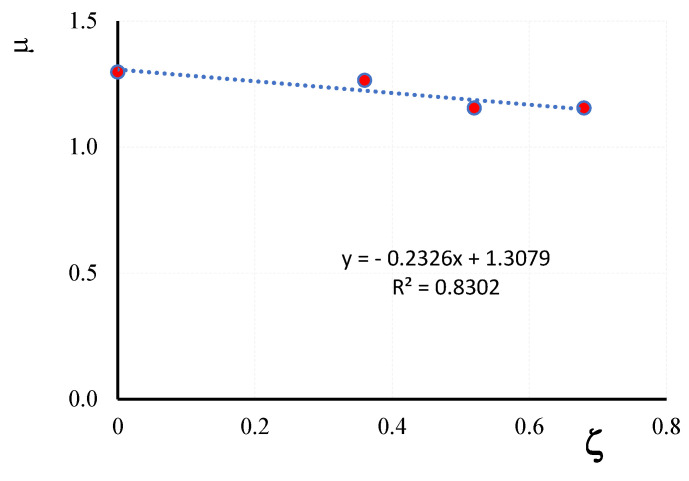
Ductility index versus vertical spacing ratio.

The ductility of the columns is measured by the ductility index *μ = ε_u_*/*ε_peak_*, defined as the ratio between the ultimate axial strain *ε_u_*, conventionally set equal to the strain value corresponding to the 85% of the peak strength on the descending branch of the axial stress-axial strain curves and the axial strains values corresponding to the peak loads, *ε_peak_*. For the tested columns, the *μ* values reported in Table 3 evidence that the ductility of the confined columns is not much different from that of unconfined ones. The ratio *δ = μ_conf_*/*μ_unconf_*, being *μ_conf_* and *μ_unconf_* the average value of the ductility index of confined and unconfined columns, respectively, varies in fact between 0.96 for CD1L-PBO3 columns and 1.05 for CD1LPBO5; for fully wrapped columns *δ =* 1.08. The variation of the ductility index μ with the vertical spacing ratio ζ is illustrated in Figure 12; it also appears that the *μ*-*ζ* law can be represented by a linear trend line (*R*^2^ = 0.83).

The ductility index of partially confined columns was reduced with respect to the values obtained for fully confined columns. Considering the averaged data, it resulted in reductions of 2.5% (CD1L-PBO5 columns), 10.9% (CD1L-PBO4 columns) and 11% (CD1L-PBO3 columns). Again, significant reductions in the amount of reinforcing fiber do not affect the ductility of the confined columns.

### 3.5. Hoop Strain

**Figure 13:**
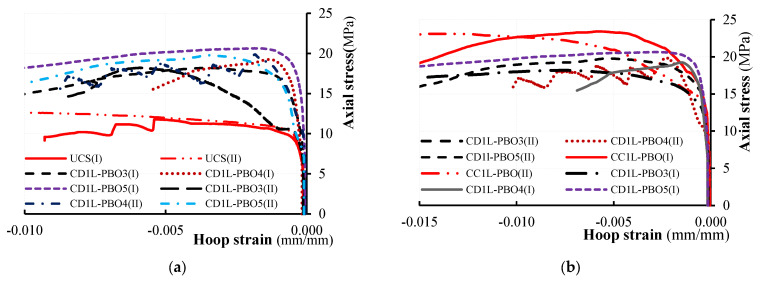
Axial stress-hoop strain curves for all tested columns: (**a**) unconfined zone and (**b**) confined zone.

As mentioned earlier, hoop strain values recorded during tests are very small in comparison with axial strain values. Hoop strains have been calculated through the measurements of lateral displacements provided by LVDTs. These measurements are reliable in the pre-cracking stage, while they are strongly affected by the presence of cracks. Consequently, the values of the hoop strain calculated in the post-peak stage are not reliable; the maximum value of hoop strains has to be considered in correspondence with the maximum stress. The comparisons between the average hoop strains measured in the unconfined and confined zones for all tested columns are reported in Figure 13.

The trend of the curves reported in Figure 13 allows us to distinguish all tested columns and three different behaviors. For low load values, the behavior of all tested columns, both un-confined and confined, is characterized by an almost linear trend of the stress–strain curves. In this phase, the columns are uncracked, and the confinement is not activated. Increasing the axial load after the cracking formation, the dilation of the masonry activates the confinement of the FRCM jacket; a progressive increase of both tensile stress in the composites and confining stress on the masonry occurs. When the FRCM jacket is fully activated (i.e., after the peak strength is reached), the confinement stress varies with the stiffness of the FRCM. However, due to the damage in the unconfined zones stage, the strength decreases as the strain increases (softening phase).

The analysis of the hoop strains variation in the un-confined (Figure 13a) and confined (Figure 13b) zones evidences a different behavior of partially confined columns as the vertical spacing ratio changes. For the highest value of the vertical spacing ratio (CD1L-PBO3 columns), after the cracking formation, the slope of the axial stress-hoop strain curves is less pronounced with respect to that of the other curves. This is an expected result because it depends on the damage produced by cracking, which, as described earlier, is more prevalent in columns where the unconfined zones are larger.

**Figure 14:**
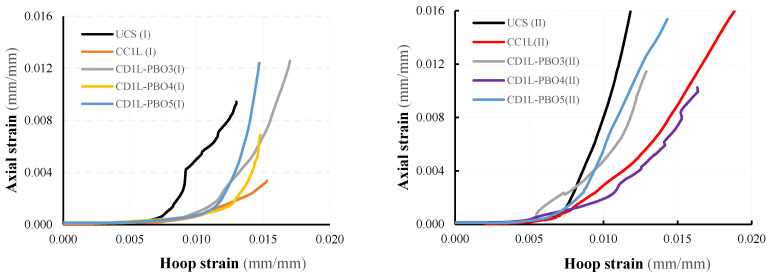
Axial strain-hoop strain curves.

Figure 14 shows the variation of the average hoop strain, measured in correspondence with the confined zones, with the average axial strain of the specimens. It is observed that such variation is linear for all the specimens tested for low values of the stress; then, following the cracking of the masonry and the activation of the confinement by the fibers, the variation becomes non-linear with the increasing slope with the applied load.

It is also noted that in the unconfined columns, the non-linear trend of the curve was manifested for load levels lower than those corresponding to the beginning of the non-linear portion of the confined specimens.

The maximum values of the hoop strain determined from lateral displacements measured by LVDTs positioned on unconfined (*ε_hmas_*) and confined (*ε_hFRCM_*) zones are summarized in Table 4 for each tested column. The exam of the values reported in Table 4 shows that for some pairs of columns, the measured values are significantly different from each other (for example, in the unconfined UCS (I) and UCS (II) columns); this is due to the presence of cracks formed in the measurement zone. Moreover, the values of the confined part (i.e., on the FRCM strips) are, on average, higher than those measured on the unconfined part in most columns. It should still be noted that the hoop strains are decreasing with the ζ-ratio both in unconfined and confined zones. For the confined columns, the values of the exploitation ratio ε_hFRCM_/ε_fu_ being ε_fu,_ the ultimate strain of the PBO fibers, are also reported in Table 4.

The exploitation ratio allows for the evaluation of the potential confining action of the FRCM jackets; in the tested columns, except for the fully wrapped CC1L-PBO (II) column for which the exploitation ratio was 0.867, the obtained values are lesser than 0.5. In particular, the average value of the exploitation ratio was 0.43 for CD1L-PBO3 (I) and CD1L-PBO3 (II) columns, while significantly lower values were obtained for CD1L-PBO4 (average of 0.125) and CD1L-PBO5 (average of 0.20). Therefore, the potential FRCM confining effectiveness did not seem to be used at all, especially for low values of the vertical spacing ratio.

## 4. Design Consideration

The above-described experimental results evidence the role of the confinement configuration represented by the vertical spacing ratio on the axial response of masonry columns partially confined with FRCM composites. Currently, the analytical models proposed to predict the axial strength of the FRCM confined columns refer to fully wrapped configurations. Even if derived from different approaches, prediction models present a non-linear form and differences between them in evaluating the effective confining pressure. Some proposed models are available in the technical literature [14,19,20,32,33]. In the following, the Italian CNR DT-215 and the American ACI 549.6R-20 guidelines are used for comparison with experimental results. The Italian Guidelines CNR-DT 215 [25] allow us to predict the confinement ratio as follows:

(1) $$\frac{f_{mc}}{f_{m0}}=1+k{\left(\frac{f_{l,eff}}{f_{m0}}\right)}^{0.5}$$

where *f_mc_* and *f_m_*_0_, are the compression strength of the confined and un-confined columns, respectively, *f_l,eff_* the effective confining pressure expressed as:

(2) $$f_{l,eff}=k_{H}f_{l}$$

(3) $$\mathrm{being}\;f_{l}=\frac{2n_{f}t_{f}E_{f}\varepsilon_{fred}}{max(h,b)}\frac{b_{f}}{s_{f}^{\prime }}$$

(4) $$\mathrm{and}\;k_{H}=1-\frac{{\left(b-2r\right)}^{2}+{\left(h-2r\right)}^{2}}{3bh}$$

where *n_f_* is the number of FRCM layers, *t_f_* is the thickness of the fiber mesh (mm), *b* and *h* are the length (mm) and the width (mm) of the cross-section of the column, *r* is the radius of the rounding corner of the column cross-section (mm). In Equation (1), the coefficient *k* is expressed as *k = α*_2_ (*g_m_*/1000)*^α^*^3^ being *g_m_* the masonry mass density in units of kg/m^3^ (approximately 1600 kg/m^3^ for the specimens in this study), *α*_2_ and *α*_3_ coefficients equal to 1 if further experimental data are not available, while the tensile strain of fibers is expressed as:

(5) $$\varepsilon_{fred}=\min\left[k_{mat}\eta_{a}\frac{\varepsilon_{fu}}{\gamma_{m}};0.004\right]$$

being *η_a_* and *η_m_* environmental and partial safety factors (taken as 1.0 in this study),

(6) $$k_{mat}=1.81{\left(\rho_{mat}f_{cmat}/f_{m0}\right)}^{2}$$

(7) $$\rho_{mat}=4t_{mat}/D$$

where *f_cmat_* and *t_mat,_* the compressive strength and the thickness of the mortar, respectively.

The ACI 549-R20 guideline [26] furnishes the following relationships to predict the confinement ratio of the FRCM confined masonry columns:

(8) $$\frac{f_{mc}}{f_{m0}}=1+3.1k_{a}\left(\frac{f_{l}}{f_{m0}}\right)$$

Being:

(9) $$k_{a}=\frac{A_{e}}{{A_{c}}_{}}{\left(\frac{b}{h}\right)}^{2}$$

(10) $$f_{l}=\frac{2n_{f}t_{f}E_{f}\varepsilon_{fe}}{D}$$

(11) $$\frac{A_{e}}{A_{c}}=1-\frac{\left(\frac{b}{h}\right){\left(b-2r\right)}^{2}+\left(\frac{h}{b}\right){\left(h-2r\right)}^{2}}{3bh}$$

(12) $$\varepsilon_{fe}=\varepsilon_{fd}<0.012$$

Both guidelines refer to masonry columns fully wrapped with FRCM jackets along their entire height; no provisions are given for partially confined masonry columns. However, in order to take partial confinement into account, it may be useful to introduce the “vertical coefficient of efficiency” in Equations (1) and (8):

(13) $$k_{v}={\left(1-\frac{s_{f}^{\prime }}{2d_{min}}\right)}^{2}$$

provided by the CNR-DT200 guidelines [9] for evaluating the strength capacity of concrete elements partially confined with FRP. In Equation (13), *d_min_* is defined and *d_min_ = min (b,h)*. Consequently, the Equations (3) and (10) become:

(14) $$\frac{f_{mc}}{f_{m0}}=1+k{\left(\frac{{k_{v}f}_{l,eff}}{f_{m0}}\right)}^{0.5}$$

(15) $$\frac{f_{mc}}{f_{m0}}=1+3.1k_{a}k_{v}\left(\frac{f_{l}}{f_{m0}}\right)$$

Table 5 reports the comparison between predictions of the CNR-DT [10] and ACI 549-R20 [11] guidelines and experimental results in terms of confinement ratio *f_mc_*/*f_m_*_0_.

The results reported in Table 5 clearly show that both of the considered guidelines provide predictions that are too conservative. A more accurate formulation of the vertical coefficient of efficiency is, then, necessary to consider the different performances of the FRCM confinement systems with respect to the FRP counterparts.

## 5. Conclusions

The paper describes the experimental behavior of clay brick masonry columns partially confined with PBO FRCM jackets. Compression tests have been executed on small-size masonry columns, varying the vertical spacing ratio considered a key parameter in the partial confinement mechanism. A comparison between the experimental data in terms of the confinement ratio and predictions of models provided by Italian CNR-DT 215 and American ACI 549-4R20 guidelines has also been made. The obtained results show the following:

- The FRCM partial confinement has favorable effects on both axial strength and axial strain of masonry columns;
- The values of the vertical spacing ratio influenced the structural response of partially confined columns; the axial capacity of confined columns was, in fact, decreasing with the increase of this ratio;
- The values of the confinement ratio ξ increase as the vertical spacing ratio decreases up to fully wrapped, varying between 1.49–1.91;
- The partially confined columns exhibited a post-peak branch that evidenced a dissipation capacity and load retention;
- The ductility index of confined columns is not much different from that of unconfined columns; its value varies between 1.07 and 1.31;
- Theoretical predictions were too conservative for all tested specimens; from these results emerged the need for more accurate formulations based on an adequate evaluation of the vertical coefficient of efficiency. The relation defined for FRP confined columns (Equation (13)) was inadequate to predict the behavior of FRCM masonry columns.

Further experimental investigations are needed for a more accurate analysis of the influence of the confinement configuration on the structural response of partially confined columns. At the same time, the definition of appropriate analytical relationships that can predict the axial strength of the columns must be based on both geometric parameters, such as the vertical spacing ratio and mechanical parameters related to the FRCM confinement system.

## Figures and Tables

**Table 1:** Geometrical details of tested columns.

Specimen Designation	Type of Confinement	*b_f_* (mm)	*s^’^_f_* (mm)	*s_f_* (mm)	ζ = *s_f_’*/*s_f_* (-)	*s^’^_f_*/*b_f_* (-)	*V_f_*/*V*_*f*0_
UCS (I)	–	–	–	–	–	–	-
UCS (II)	–	–	–	–	–	–	-
CC1L-PBO (I)	Continuous	445	0.0	–	0	0	1.0
CC1L-PBO (II)	Continuous	445	0.0	–	0	0	1.0
CD1L-PBO3 (I)	Discontinuous	55	120.0	135.0	0.89	2.18	0.32
CD1L-PBO3 (II)	Discontinuous	55	120.0	135.0	0.89	2.18	0.32
CD1L-PBO4 (I)	Discontinuous	55	61.7	116.7	0.53	1.12	0.48
CD1L-PBO4 (II)	Discontinuous	55	61.7	116.7	0.53	1.12	0.48
CD1L-PBO5 (I)	Discontinuous	55	32.5	87.5	0.37	0.59	0.60
CD1L-PBO5 (II)	Discontinuous	55	32.5	87.5	0.37	0.59	0.60

**Table 2:** Mechanical properties of the mortars.

Label	Unit	Mortar 1 (MPa)	Mortar 2 (MPa)	Mortar 3 (MPa)
*f_b,mat_* (CoV)	(MPa)	1.9 (4%)	8.6 (3%)	4.5 (6%)
*f_c,mat_* (CoV)	(MPa)	5.2 (4%)	34.1(3%)	24.8 (8%)

**Table 3:** Test results.

Specimen Designation	Axial Peak Load (kN)	Average Axial Peak Load (%Scatter) (kN)	Confinement Ratio *ξ* (-)	Axial Peak Strain *ε_peak_* (-)	Ultimate Axial Strain *ε_u_* (-)	Ductility Index μ (-)	Elastic Modulus *E_mas_* (MPa)
UCS (I)	737.16	765.45 (7.1)	-	0.01134	0.01368	1.206	1110.70
UCS (II)	793.75		-	0.01488	0.01780	1.196	1441.10
CC1L-PBO (I)	1463.76	1453.76 (1.4)	1.910	0.01853	0.0237	1.279	1749.50
CC1L-PBO (II)	1443.75		1.886	0.02299	0.02768	1.314	2122.70
CD1L-PBO3 (I)	1137.48	1137.52 (0.0)	1.486	0.01532	0.01892	1.235	1457.50
CD1L-PBO3 (II)	1137.56		1.486	0.01426	0.01530	1.073	1581.10
CD1L-PBO4 (I)	1203.76	1223.71 (3.2)	1.572	0.01353	0.01566	1.157	1570.40
CD1L-PBO4 (II)	1243.67		1.625	0.01494	0.01718	1.150	1816.20
CD1L-PBO5 (I)	1290.36	1263.94 (4.1)	1.686	0.01355	0.01702	1.256	1616.70
CD1L-PBO5 (II)	1237.52		1.617	0.01317	0.01676	1.272	2093.30

**Table 4:** Test results: hoop strain values.

Specimen Designation	Hoop Strain in Confined Zone *ε_hFRCM_* (mm/mm)	Hoop Strain in Unconfined Zone *ε_hmas_* (mm/mm)	Exploitation Ratio *ε_hFRCM_*/*ε_fu_* (-)
UCS (I)	-	0.05584	-
UCS (II)	-	0.01135	-
CC1L-PBO (I)	0.00567	-	0.363
CC1L-PBO (II)	0.01347	-	0.863
CD1L-PBO3 (I)	0.00700	0.00400	0.449
CD1L-PBO3 (II)	0.00642	0.00588	0.411
CD1L-PBO4 (I)	0.00149	0.00130	0.096
CD1L-PBO4 (II)	0.00230	0.00184	0.147
CD1L-PBO5 (I)	0.00115	0.00185	0.074
CD1L-PBO5 (II)	0.00500	0.00300	0.321

**Table 5:** Comparison between the analytical and experimental results.

Specimen	Equation (14) CNR-DT215	Equation (15) ACI 549-R20	Pred/Exp CNR-DT215	Pred/Exp ACI549-R20	Experimental
CC1L-PBO (I)	1.0575	1.0093	0.5537	0.5282	1.910
CC1L-PBO (II)	1.0575	1.0093	0.5607	0.5350	1.886
CD1L-PBO3 (I)	1.0352	1.0052	0.6966	0.6764	1.486
CD1L-PBO3 (II)	1.0352	1.0052	0.6966	0.6764	1.486
CD1L-PBO4 (I)	1.0566	1.0069	0.6721	0.6405	1.572
CD1L-PBO4 (II)	1.0566	1.0069	0.6502	0.6196	1.625
CD1L-PBO5 (I)	1.0832	1.0078	0.6425	0.5978	1.686
CD1L-PBO5 (II)	1.0832	1.0078	0.6425	0.6233	1.617

## Data Availability

The data presented in this study are available on request from the corresponding author.

## References

[1] Micelli F., Aiello M.A. (2019). Residual tensile strength of dry and impregnated reinforcement fibres after exposure to alkaline environments. Compos. Part B.

[2] Arboleda D., Babaeidarabad S., Hays C.D.L., Nanni A. Durability of Fabric Reinforced Cementitious Matrix (FRCM) Composites. Proceedings of the 7th International Conference on FRP Composites in Civil Engineering.

[3] Gunes M.E., Pekmezci B.Y., Girgin Z.C. (2020). Durability of natural hydraulic lime (NHL) based TRM composite through hot water immersion method. Mater. Struct..

[4] Donnini J. (2019). Durability of glass FRCM systems: Effect of different environments on mechanical properties. Compos. Part B.

[5] https://www.ruregold.com.

[6] Chen Z., Aziz T., Sun H., Ullah A., Ali A., Cheng L., Ullah R., Khan F.U. (2023). Advances and Applications of Cellulose Bio-Composites in Biodegradable Materials. J. Polym. Environ..

[7] Aziz T., Farid A., Haq F., Kiran M., Ullah N., Faisal S., Ali A., Khan F.U., You S., Bokhari A. (2023). Role of silica-based porous cellulose nanocrystals in improving water absorption and mechanical properties. Environ. Res..

[8] Minafò G., La Mendola L. (2018). Experimental investigation on the effect of mortar grade on the compressive behaviour of FRCM confined masonry columns. Comp. Part B Eng..

[9] Fossetti M., Minafò G. (2017). Comparative experimental analysis on the compressive behaviour of masonry columns strengthened by FRP, BFRCM or steel wires. Comp. Part B Eng..

[10] Wang J., Wan C., Shen L., Zeng Q., Ji X. (2023). Compressive behavior of masonry columns confined with basal textile-reinforced concrete. J. Build. Eng..

[11] Cascardi A., Micelli F., Aiello M.A. (2018). FRCM-confined masonry columns: Experimental investigation on the effect of the inorganic matrix properties. Constr. Build. Mater..

[12] Di Ludovico M., Cascardi A., Balsamo A., Aiello M.A. (2020). Uniaxial experimental tests on full-scale limestone masonry columns confined with glass and basalt FRCM systems. J. Comp. Constr..

[13] Koutas L.N., Bournas A.D. (2020). Confinement of masonry columns with textile-reinforced mortar jackets. Constr. Build. Mater..

[14] Krevaikas T.D. (2019). Experimental study on carbon fiber textile reinforced mortar system as a means for confinement of masonry columns. Constr. Build. Mater..

[15] Ombres L. (2014). Confinement effectiveness in eccentrically loaded masonry columns strengthened by fiber reinforced cementitious matrix (FRCM) jackets. Key Eng. Mater..

[16] Ombres L., Verre S. (2020). Analysis of the behavior of FRCM confined clay brick masonry columns. Fibers.

[17] Sneed L.H., Carloni C., Baietti G., Fraioli G. (2017). Confinement of clay masonry columns with SRG. Key Eng. Mater..

[18] Aiello M.A., Bencardino F., Cascardi A., D’Antino T., Fagone M., Frana I., La Mendola L., Lignola G.P., Mazzotti C., Micelli F. (2021). Masonry columns confined with fabric reinforced cementitious matrix (FRCM) systems: A round robin test. Constr. Build. Mater..

[19] Napoli A., Realfonzo R. (2022). Compressive behavior of masonry columns confined with FRCM systems: Research overview and analytical proposal. J. Comp. Constr..

[20] Ameli Z., D’Antino T., Carloni C. (2022). A new predictive model for FRCM confined columns: A reflection on the composite behavior at peak stress. Constr. Build. Mater..

[21] Thamboo J. (2020). Performance of masonry columns confined with composites under compression: A state-of-art review. Constr. Build. Mater..

[22] Aiello M.A., Cascardi A., Verre S., Ombres L. (2020). Confinement of masonry columns with the FRCM-system: Theoretical and experimental investigation. Infrastructures.

[23] Ombres L., Guglielmi M., Verre S. (2022). Structural performances of clay brick masonry columns partially confined with FRCM/SRG composites. Key Eng. Mater..

[24] (2013). Guide for the Design and Construction of Externally Bonded FRP Systems for Strengthening Existing Structures.

[25] (2020). Guide for the Design and Construction of Externally Bonded Fibre Reinforced Inorganic Matrix Systems for Strengthening Existing Structures.

[26] (2020). Guide to Design and Construction of Externally Bonded Fabric-Reinforced Cementitious Matrix and Steel-Reinforced Grout Systems for Repair and Strengthening of Concrete Structures.

[27] (2011). Methods of Test for Masonry Units-Part 1: Determination of Compressive Strength.

[28] (2000). Product and System for the Protection and Repair Concrete Structures-Test Methods-Determination of Compressive Strength of Repair Mortar.

[29] (2006). Methods of Test for Mortar for Masonry Part 11: Determination of Flexural and Compressive Strength of Hardened Mortar.

[30] (2013). Textiles—Tensile Properties of Fabrics—Part 1: Determination of Compressive Strength Maximum Force and Elongation at Maximum Force Using the Strip Method.

[31] (2018). Guidelines for the Identification, Qualification and Acceptance Control of Fiber-Reinforced Composites with Inorganic Matrix (FRCM) for Strengthening of Existing Structures.

[32] Cascardi A., Longo F., Micelli F., Aiello M.A. (2017). Compressive strength of confined column with Fiber Reinforced Mortar (FRM):New-design-oriented models. Constr. Build. Mater..

[33] Ramaglia G., Lignola G.P., Fabbrocino F., Prota A. (2019). Multi-parameters mechanical modeling to derive a confinement model for masonry columns. Constr. Build. Mater..

